# Predicting the Antigenic Structure of the Pandemic (H1N1) 2009 Influenza Virus Hemagglutinin

**DOI:** 10.1371/journal.pone.0008553

**Published:** 2010-01-01

**Authors:** Manabu Igarashi, Kimihito Ito, Reiko Yoshida, Daisuke Tomabechi, Hiroshi Kida, Ayato Takada

**Affiliations:** 1 Department of Global Epidemiology, Hokkaido University Research Center for Zoonosis Control, Sapporo, Japan; 2 Department of Disease Control, Graduate School of Veterinary Medicine, Hokkaido University, Sapporo, Japan; 3 OIE Reference Laboratory for Highly Pathogenic Avian Influenza, Sapporo, Japan; University of Oxford, United Kingdom

## Abstract

The pandemic influenza virus (2009 H1N1) was recently introduced into the human population. The hemagglutinin (HA) gene of 2009 H1N1 is derived from “classical swine H1N1” virus, which likely shares a common ancestor with the human H1N1 virus that caused the pandemic in 1918, whose descendant viruses are still circulating in the human population with highly altered antigenicity of HA. However, information on the structural basis to compare the HA antigenicity among 2009 H1N1, the 1918 pandemic, and seasonal human H1N1 viruses has been lacking. By homology modeling of the HA structure, here we show that HAs of 2009 H1N1 and the 1918 pandemic virus share a significant number of amino acid residues in known antigenic sites, suggesting the existence of common epitopes for neutralizing antibodies cross-reactive to both HAs. It was noted that the early human H1N1 viruses isolated in the 1930s–1940s still harbored some of the original epitopes that are also found in 2009 H1N1. Interestingly, while 2009 H1N1 HA lacks the multiple *N*-glycosylations that have been found to be associated with an antigenic change of the human H1N1 virus during the early epidemic of this virus, 2009 H1N1 HA still retains unique three-codon motifs, some of which became *N*-glycosylation sites via a single nucleotide mutation in the human H1N1 virus. We thus hypothesize that the 2009 H1N1 HA antigenic sites involving the conserved amino acids will soon be targeted by antibody-mediated selection pressure in humans. Indeed, amino acid substitutions predicted here are occurring in the recent 2009 H1N1 variants. The present study suggests that antibodies elicited by natural infection with the 1918 pandemic or its early descendant viruses play a role in specific immunity against 2009 H1N1, and provides an insight into future likely antigenic changes in the evolutionary process of 2009 H1N1 in the human population.

## Introduction

In April 2009, pandemic (H1N1) 2009 influenza virus (2009 H1N1) was first found in patients with febrile respiratory illness in the United States and Mexico, and has spread rapidly across the world by human-to-human transmission. On the 11th of June 2009, the World Health Organization declared a global pandemic of 2009 H1N1 infection. H1N1 influenza virus caused a pandemic in 1918 (1918 H1N1) [1], and its descendant virus with highly altered antigenicity of the viral surface protein, hemagglutinin (HA) has been causing “seasonal flu” in humans.

The 2009 H1N1 resulted from genetic reassortment between the recently circulating swine H1 viruses in North America and the avian-like swine viruses in Europe [2]. Phylogenetic analysis showed that the HA gene of 2009 H1N1 was derived from the so-called “classical swine H1N1” virus, which likely shares a common ancestor with the recent human H1N1 virus [2]. Accordingly, it has been reported that the early strains of the classical swine H1N1 virus, which was first identified in North America in 1930, were antigenically similar to the prototype strain of 1918 H1N1, A/South Carolina/1/1918 (SC1918), detected from a few victims of the pandemic in 1918 [3], [4]. Since antigenic changes occur more slowly in swine than in the human population [5], HA of the classical swine H1N1 virus was antigenically highly conserved until the late 1990s [4], [6], raising the possibility that the recently emerged 2009 H1N1 may still retain an antigenic structure similar to that of SC1918 and the early isolates of its descendants.

In this study, we generated three-dimensional (3D) structures of the HA molecules of 1918 H1N1, its descendent, recent seasonal H1N1 viruses, and 2009 H1N1, and compared their antigenic structures to look for evidence for the existence of shared epitopes for neutralizing antibodies. Since the 2009 H1N1 HA antigenic sites will be targeted by antibody-mediated selection pressure in humans in the near future, we further discuss possible directions of antigenic changes in the evolutionary process of this pandemic virus.

## Results and Discussion

**Figure 1:**
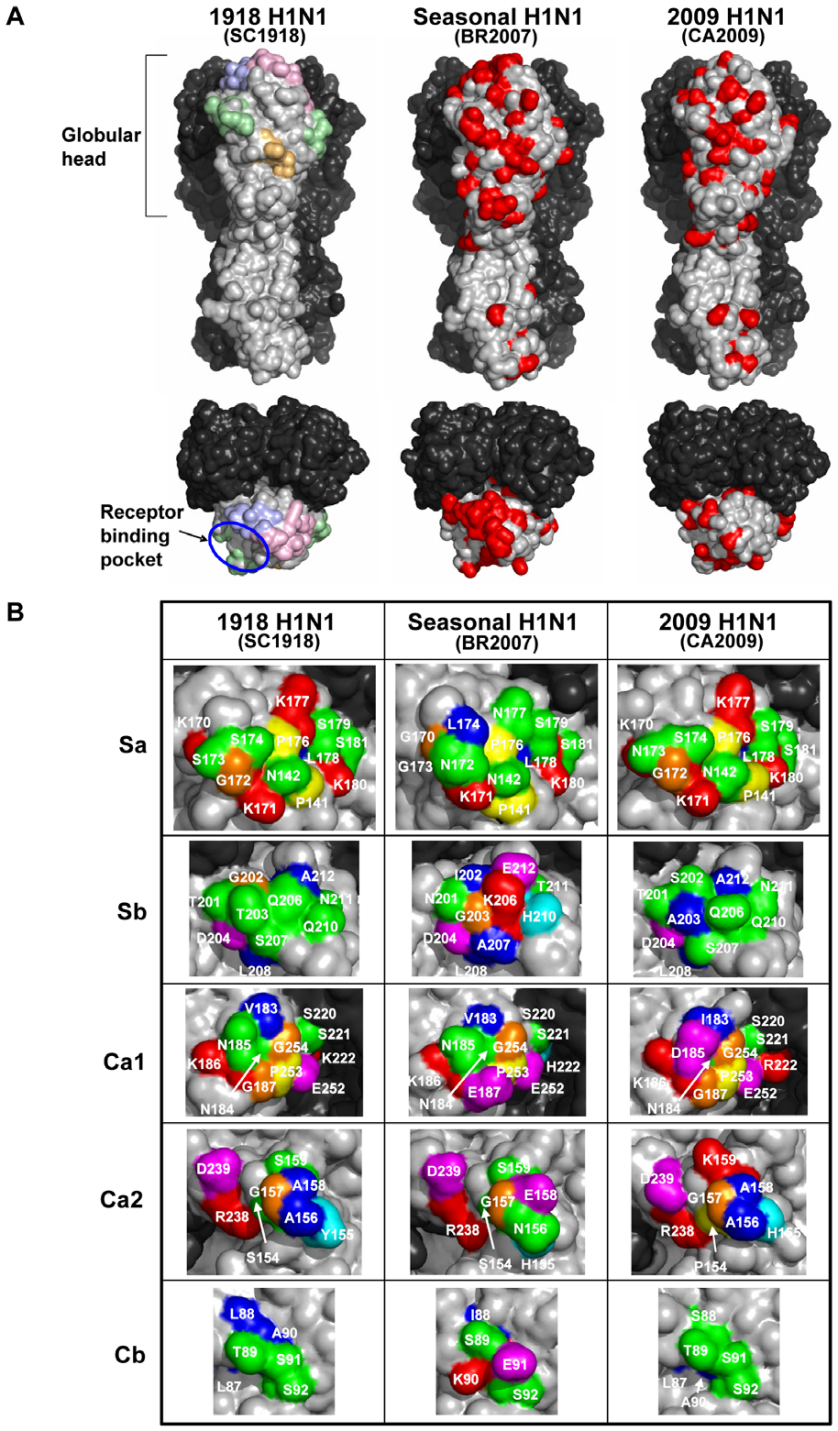
Comparison of the structures of antigenic sites on the HA molecules among 1918 H1N1 (SC1918), recent seasonal H1N1 (BR2007), and 2009 H1N1 (CA2009). Three-dimensional models of the H1 HA molecules of SC1918, BR2007, and CA2009 were constructed based on the HA crystal structures of A/South Carolina/1/18, A/Puerto Rico/8/34, and A/swine/Iowa/30, respectively (PDB codes: 1RUZ, 1RU7, and 1RUY, respectively). Models with solvent-accessible surface representation were generated by a molecular modeling method as described in the Methods section. Molecular surface of the HA trimers viewed on its side (upper) and top (lower) are shown (A). One monomer (center) is colored gray and the others are colored dark gray. The antigenic sites, Sa (light pink), Sb (light blue), Ca (pale green), and Cb (light orange) are indicated on the model of SC1918 HA. The spatial locations of amino acid residues that are distinct from those of SC1918 HA are shown in red on the models of BR2007 and CA2009 HAs. Each amino acid residue is mapped on the close-up views of each antigenic site of SC1918, BR2007, and CA2009 HAs (B). The Ca site is divided into subregions, Ca1 and Ca2. Amino acids are colored by the default ClustalX color scheme [29]: Trp, Leu, Val, Ile, Met, Phe, and Ala (blue); Lys and Arg (red); Thr, Ser, Asn, and Gln (green); Cys (pink); Asp and Glu (magenta); Gly (orange); His and Tyr (cyan); Pro (yellow).

It is known that the H1 HA molecules have four distinct antigenic sites: Sa, Sb, Ca, and Cb [7], [8], [9], [10] (Figure 1). As a result, these sites consist of the most variable amino acids in the HA molecule of the seasonal human H1N1 viruses that have been subjected to antibody-mediated immune pressure since its emergence in 1918 [3]. To investigate the structures of these antigenic sites of 2009 H1N1, 3D structures of the HA molecules of SC1918, the recent seasonal human H1N1 virus A/Brisbane/59/2007 (BR2007), and 2009 H1N1 A/California/04/2009 (CA2009) [2] were constructed by a homology modeling approach, and compared by mapping all the amino acid residues that were distinct from those of SC1918 HA (Figure 1 and Table S1). We found that most of these antigenic sites of BR2007 HA predominantly contained altered amino acid residues if compared with SC1918. By contrast, amino acid residues at these positions were relatively conserved in CA2009 HA. Notably, the Sa and Sb sites that contain many amino acids involved in neutralizing epitopes near the receptor binding pockets [8], [10] remain almost intact in CA2009 HA (Table 1), suggesting that antibodies raised by natural infection with SC1918 or its antigenically related descendant viruses play a role in specific immunity against CA2009.

**Table 1:** Amino acid similarity in the HA antigenic sites among recent seasonal H1N1 (BR2007), 2009 H1N1 (CA2009), and 1918 H1N1 (SC1918).

Antigenic sites	No. amino acids involved	No. of amino acids identical to SC1918	
		BR2007	CA2009
Sa	13	8	12
Sb	12	4	10
Ca	19	13	13
Cb	6	2	5

**Figure 2:**
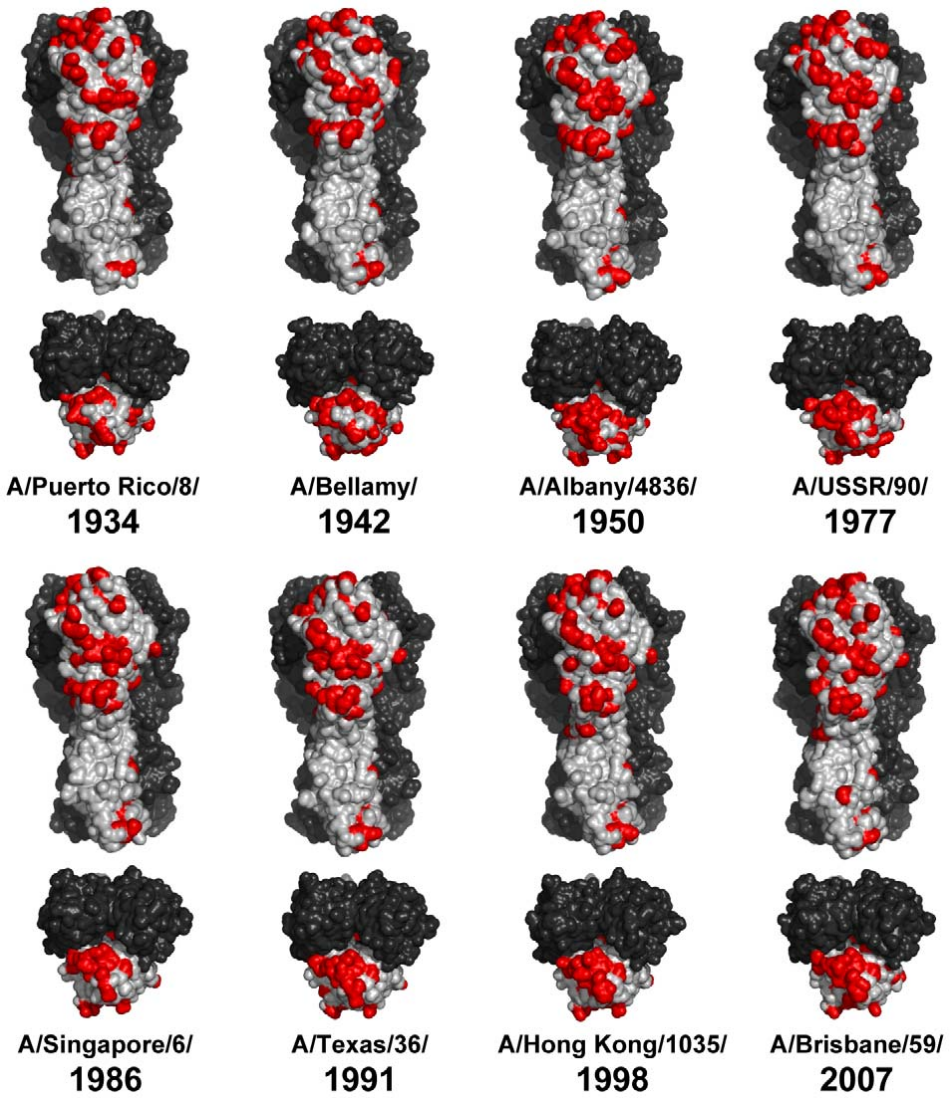
Amino acid substitutions associated with antigenic changes of seasonal human H1N1 virus HAs. All models were generated and shown by a molecular modeling method as described in the Methods section and the legend of Figure 1.

We then constructed 3D structures of the representative strains of seasonal H1 viruses that had been isolated since 1934, and tracked the amino acid substitutions on their HA molecules (Figure 2 and Figure S1). We confirmed that amino acid substitutions associated with the antigenic changes gradually accumulated on the globular head region of HA and were distributed over four distinct antigenic sites. However, it was noted that the early isolates represented by the A/Puerto Rico/8/1934 and A/Bellamy/1942 strains, but not the strains isolated after the 1950s, still harbored unchanged amino acids forming potential neutralizing epitopes in the Sa and Sb sites (Figure 2). It seems likely that most of the amino acids on these antigenic sites were eventually substituted in the late 1940s (Figure S1).

**Figure 3:**
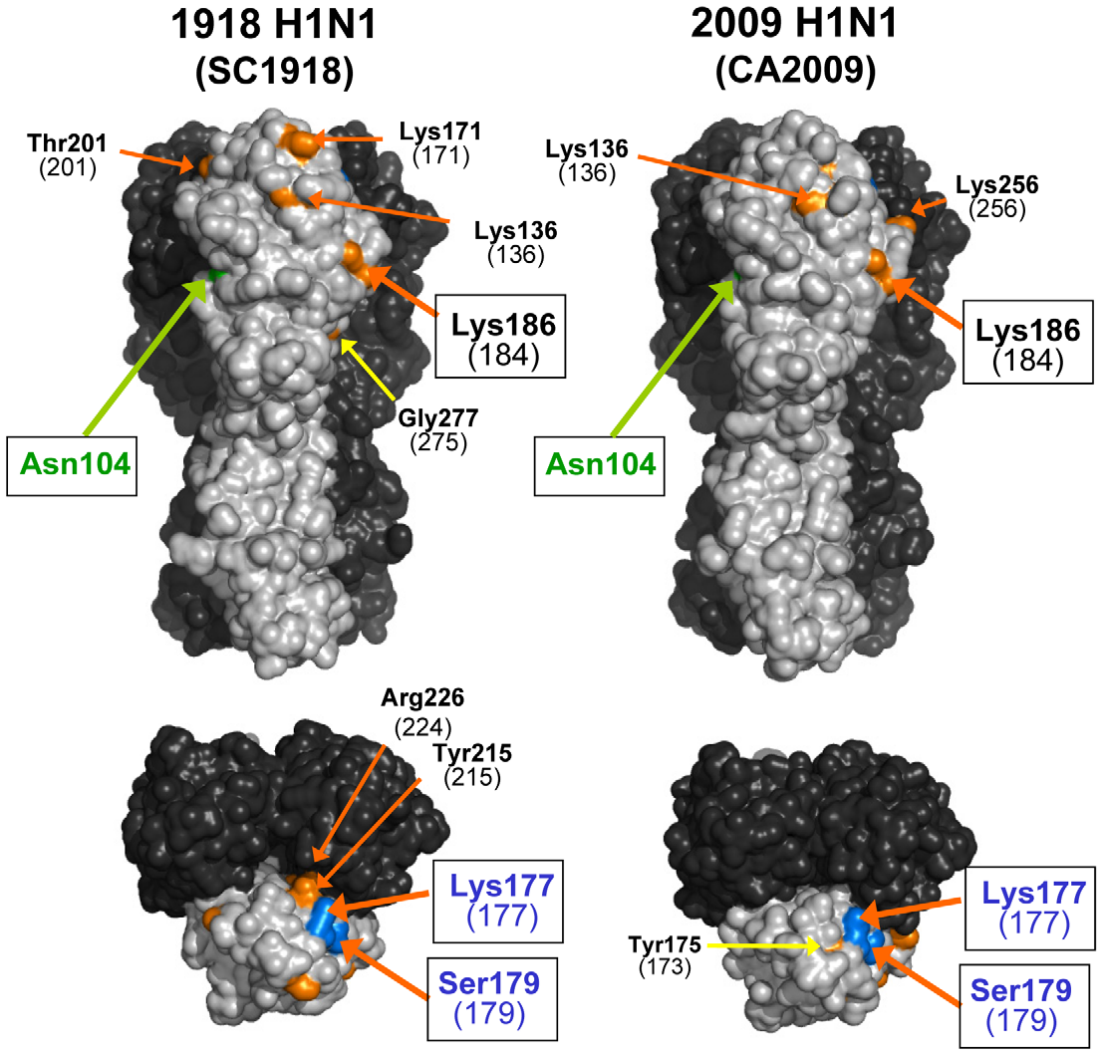
Comparison of the *N*-glycosylation potential of HA between SC1918 and CA2009. Residues shown in green represent Asn at the actually existing *N*-glycosylation sites. Residues shown in orange or blue represent the amino acids in *Cand1* sites that require a nucleotide substitution to produce *N*-glycosylation sites. Residues shown in blue represent the amino acids that were actually substituted, resulting in the acquisition of *N*-glycosylation sites during the antigenic evolution of human H1N1 viruses. Numbers in parentheses show the positions of Asn residues that may be linked to carbohydrate chains, if respective *Cand1* sites mutate to have *N*-glycosylation sites. All models were generated as described in the Methods section and the legend of Figure 1.

It is well-documented that antigenic changes of HA occasionally result in the acquisition of carbohydrate side chains on the HA molecule [8], [11]. Since the carbohydrate side chains in the vicinity of antigenic sites mask the neutralizing epitopes on the HA surface, amino acid substitutions associated with acquisition of carbohydrate chains are believed to efficiently generate antigenic variants. Accordingly, recent seasonal H1N1 viruses have acquired 4–5 *N*-glycosylation sites (Asn-Xaa-Ser/Thr, where Xaa is any amino acid except Pro) in the globular head region of HA [12], [13], whereas SC1918 HA had only one site, at Asn 104 (Figure 3).

Interestingly, CA2009 also has a single potential *N*-glycosylation site at the same position in the globular head region of HA (Figure 3), despite the fact that the classical swine H1N1 virus emerged in the early 1900s and was circulating in the pig population until recently. This prompted us to estimate the potential of 2009 H1N1 to acquire additional *N*-glycosylation sites on its HA, which may be related to its future evolutionary process in the human population. We previously defined a three-codon motif that becomes an *N*-glycosylation site with a single-nucleotide mutation as “*Cand1*”, and suggested that the presence of the *Cand1* sites in the HA sequence is one of the key factors for human influenza A viruses to rapidly acquire *N*-glycosylation sites during the early epidmic in the human population [13]. We compared the number of the *Cand1* sites in the HA globular head region between SC1918 and CA2009 (Figure 3 and Table S1). We found that CA2009 HA possessed three *Cand1* sites on the antigenic sites Sa and Ca, all of which were also present at the same position in SC1918 HA (positions of the first Asn residue, 177, 179, and 184). Of these, the *Cand1* sites with positions at 177 and 179 had actually become potential *N*-glycosylation sites in human H1N1 viruses, although these two sites did not exist concurrently [12]. It is noted that these two *Cand1* sites are still present on the surface of CA2009 HA, suggesting the likelihood of additional *N*-glycosylation at these sites during future antigenic changes of 2009 H1N1 HA.

In this paper, we employed 3D structures constructed by a homology modeling method to map amino acid residues on the antigenic sites of HA. When compared to the presentation of simple primary sequences, the 3D presentation has following advantages: (a) There are several amino acid residues that are buried beneath the surface of the HA molecule, even if they are included in the antigenic sites described by the primary amino acid sequences. Since such amino acid residues do not directly contribute to the interaction with antibodies, the surface structures of antigenic sites that are accessible for antibodies can be compared more precisely in the presentation by 3D models than by the primary amino acid sequence. (b) An epitope likely consists of multiple amino acid residues belonging to different antigenic regions presented by the primary amino acid sequence. Such conformational epitopes can be illustrated only by the 3D presentation. (c) One of the purposes of this study is to provide a structural basis to confirm antigenic similarity between the 1918 H1N1 and the pandemic 2009 H1N1 viruses. For this purpose, we employed a homology modeling method rather than simply mapping on the existing crystal structure (e.g. 1918 H1N1 HA), since this method is generally used to generate a 3D structure of a protein molecule if there is no available crystal structure of the target protein [14]. Thus, we believe that this method produces more likely HA structure models of the viruses whose HA crystal structure are not available (e.g. CA2009). In fact, our homology modeling approach suggests that several amino acid residues were occasionally buried beneath or exposed to the surface of HA molecule, depending on the substitutions found in the viruses examined (Figure 1B and Figure S1). The homology modeling approach might enable us to analyze such dynamics of antigenic changes at molecular levels.

Our analysis indicated that 2009 H1N1 had undergone less significant antigenic changes of HA in the pig population than human H1N1 virus since their emergence in the early 1900s. The Centers for Disease Control and Prevention reported that vaccination with recent (2005–2009) human H1N1 viruses was unlikely to provide protection against 2009 H1N1 [15]; however, cross-reactive antibodies were detected in 33% of people aged 60 and over. Another report showed that appreciable neutralizing antibodies against CA2009 were present in the sera collected from individuals born before 1918 [16]. Our 3D models provide a protein-structural basis supporting these observations, and further suggest that infection with the 1918 H1N1 or early human H1N1 viruses (viruses present before the 1940s), but not with antigenically divergent human H1N1 viruses circulating after the 1950s, elicited cross-neutralizing antibodies to 2009 H1N1.

**Figure 4:**
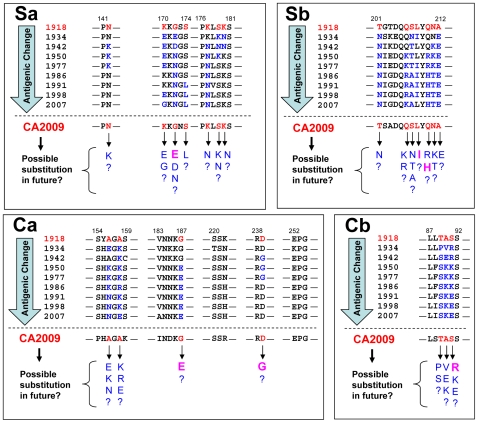
Prediction of the future amino acid substitutions on the antigenic sites of 2009 H1N1 HA. Amino acid sequences of HA antigenic sites of human H1N1 viruses are shown. Sequence data are corresponding to those of virus strains shown in Figures 1 and 2. Amino acid residues shared between 1918 H1N1 (SC1918) and 2009 H1N1 (CA2009) are shown in red, and those that have been substituted since 1934 are shown in blue. Amino acid residues indicated by arrows represent the predicted substitutions which might be associated with antigenic changes of 2009 H1N1 in the near future. The amino acid substitutions which have already been found in the recent variants of the 2009 H1N1 virus (as of November 3, 2009) are shown in bold pink letters.

This virus will soon be subjected to complex immunological selection pressure by the antibody response that will be induced in the human population by vaccination and/or natural infection with homologous viruses, and pre-existing immunity cross-reactive to the early descendants of 1918 H1N1. In the present study, we showed that the antigenic structure of 2009 H1N1 HA might still be similar, at least in part, to that of the 1918 H1N1 HA. We speculate that the 2009 H1N1 HA antigenic sites involving the conserved amino acids will soon be targeted by neutralizing antibodies in humans. Thus, it is of interest to monitor whether these antigenic sites of 2009 H1N1 will undergo similar patterns of amino acid substitutions to those seen in seasonal H1N1 viruses during its epidemic period (Figure 4). Interestingly, we found that some of the recent variants of the 2009 H1N1 virus (as of November 3, 2009) have indeed undergone substitutions identical to those predicted in Figure 4. Although the present study still needs to be supported by experimental data, our approach may provide new perspectives on collective immunity against 2009 H1N1 and an insight into future antigenic changes of this new human pandemic influenza virus.

## Methods

### Sequence Data of HA Genes

Nucleotide sequences for HA genes of SC1918 (AF117241), BR2007 (CY030230), CA2009 (FJ966082), A/Puerto Rico/8/1934/Mount Sinai (AF389118), A/Bellamy/1942 (CY009276), A/Albany/4836/1950 (CY021701), A/USSR/90/1977 (DQ508897), A/Singapore/6/1986 (CY020477), A/Texas/36/1991 (AY289927), and A/Hong Kong/1035/1998 (AF386777) [2], [3], [17], [18], [19], [20] were obtained from Influenza Virus Resource at the National Center for Biotechnology Information (NCBI) (http://www.ncbi.nlm.nih.gov/genomes/FLU/FLU.html).

### Molecular Modeling

MODELLER 9v6 [21] was used for homology modeling of HA structures. After one hundred models of the HA trimer were generated, the model was chosen by a combination of the MODELLER objective function value and the discrete optimized protein energy (DOPE) statistical potential score [22]. After addition of hydrogen atoms, the model was refined by energy minimization (EM) with the minimization protocols in the Discovery Studio 2.1 software package (Accelrys, San Diego, CA) using a CHARMm force field. Steepest descent followed by conjugate gradient minimizations was carried out until the root mean square (rms) gradient was less than or equal to 0.01 kcal/mol/Å. The generalized Born implicit solvent model [23], [24] was used to model the effects of solvation. The HA model was finally evaluated by using PROCHECK [25], WHATCHECK [26], and VERIFY-3D [27]. All figures are shown as a solvent-accessible surface representation prepared by PyMOL (DeLano Scientific LLC) [28]. All HA structures constructed by a homology modeling method are available in Supplementary Files S1, S2, S3, S4, S5, S6, S7, and S8.

### Sequence Data Analyses for *N*-Glycosylation Sites

Custom-made programs were developed with the Ruby language and used for investigating the numbers of potential *N*-glycosylation sites and candidate codons (*Cand1*) in HA sequences. The programs are available upon request.

## Supporting Information

Table S1 (0.04 MB PDF)

Figure S1 Amino acid substitutions of seasonal human H1N1 virus HAs shown in close-up views of each antigenic site. The strains used in this analysis are corresponding to those shown in Figure 2. Amino acids are colored according to the scheme in the legend of Figure 1B.
(1.02 MB PDF)

File S1 PDB file of the homology model of H1 HA (A/California/04/2009) after energy minimizations.
(0.20 MB ZIP)

File S2 PDB file of the homology model of H1 HA (A/Bellamy/1942) after energy minimizations.
(0.20 MB ZIP)

File S3 PDB file of the homology model of H1 HA (A/Albany/4836/1950) after energy minimizations.
(0.20 MB ZIP)

File S4 PDB file of the homology model of H1 HA (A/USSR/90/1977) after energy minimizations.
(0.20 MB ZIP)

File S5 PDB file of the homology model of H1 HA (A/Singapore/6/1986) after energy minimizations.
(0.20 MB ZIP)

File S6 PDB file of the homology model of H1 HA (A/Texas/36/1991) after energy minimizations.
(0.20 MB ZIP)

File S7 PDB file of the homology model of H1 HA (A/Hong Kong/1035/1998) after energy minimizations.
(0.20 MB ZIP)

File S8 PDB file of the homology model of H1 HA (A/Brisbane/59/2007) after energy minimizations.
(0.20 MB ZIP)
