# Supplementary material for: Predicting the Antigenic Structure of the Pandemic (H1N1) 2009 Influenza Virus Hemagglutinin
Source: PLoS One. 2010 Jan 1;5(1):e8553. doi: 10.1371/journal.pone.0008553 (PMC2797400; doi:10.1371/journal.pone.0008553)
Supplement: Table S1 — (0.04 MB PDF) [file pone.0008553.s001.pdf]

Table S1. Individual data for amino acid residues in HA

Region	Amino acid position	SC1918		BR2007		CA2009		Antigenic site	Contribution to N-glycosylation
		Residue	Codon	Residue	Codon	Residue	Codon		
Signal peptide	1	M	ATG	M	ATG	M	ATG		
Signal peptide	2	E	GAG	K	AAA	K	AAG		
Signal peptide	3	A	GCA	V	GTA	A	GCA		
Signal peptide	4	R	AGA	K	AAA	I	ATA		
Signal peptide	5	L	CTA	L	CTA	L	CTA		
Signal peptide	6	L	CTG	L	CTG	V	GTA		
Signal peptide	7	V	GTC	V	GTC	V	GTT		
Signal peptide	8	L	TTG	L	CTG	L	CTG		
Signal peptide	9	L	TTA	L	TTA	L	CTA		
Signal peptide	10	C	TGT	C	TGC	Y	TAT		
Signal peptide	11	A	GCA	T	ACA	T	ACA		
Signal peptide	12	F	TTT	F	TTT	F	TTT		
Signal peptide	13	A	GCA	T	ACA	A	GCA		
Signal peptide	14	A	GCT	A	GCT	T	ACC		
Signal peptide	15	T	ACA	T	ACA	A	GCA		
Signal peptide	16	N	AAT	Y	TAT	N	AAT		
Signal peptide	17	A	GCA	A	GCA	A	GCA		
HA1	18	D	GAC	D	GAC	D	GAC		
HA1	19	T	ACA	T	ACA	T	ACA		
HA1	20	I	ATA	I	ATA	L	TTA		
HA1	21	C	TGT	C	TGT	C	TGT		
HA1	22	I	ATA	I	ATA	I	ATA		
HA1	23	G	GGC	G	GGC	G	GGT		
HA1	24	Y	TAC	Y	TAC	Y	TAT		
HA1	25	H	CAT	H	CAT	H	CAT		
HA1	26	A	GCG	A	GCT	A	GCG		
HA1	27	N	AAT	N	AAC	N	AAC		sequon(All)
HA1	28	N	AAC	N	AAC	N	AAT		sequon(All)
HA1	29	S	TCA	S	TCG	S	TCA		
HA1	30	T	ACC	T	ACC	T	ACA		Cand1(SC1918), Cand1(BR2007)
HA1	31	D	GAC	D	GAC	D	GAC		
HA1	32	T	ACT	T	ACT	T	ACT		
HA1	33	V	GTT	V	GTT	V	GTA		
HA1	34	D	GAC	D	GAC	D	GAC		
HA1	35	T	ACA	T	ACA	T	ACA		
HA1	36	V	GTA	V	GTA	V	GTA		
HA1	37	L	CTC	L	CTT	L	CTA		
HA1	38	E	GAA	E	GAA	E	GAA		
HA1	39	K	AAG	K	AAG	K	AAG		
HA1	40	N	AAT	N	AAT	N	AAT		sequon(All)
HA1	41	V	GTG	V	GTG	V	GTA		
HA1	42	T	ACC	T	ACA	T	ACA		Cand1(SC1918)
HA1	43	V	GTG	V	GTG	V	GTA		
HA1	44	T	ACA	T	ACA	T	ACA		
HA1	45	H	CAC	H	CAC	H	CAC		
HA1	46	S	TCT	S	TCT	S	TCT		
HA1	47	V	GTT	V	GTC	V	GTT		
HA1	48	N	AAC	N	AAC	N	AAC		
HA1	49	L	CTG	L	CTG	L	CTT		
HA1	50	L	CTC	L	CTT	L	CTA		
HA1	51	E	GAA	E	GAG	E	GAA		
HA1	52	D	GAC	N	AAC	D	GAC		
HA1	53	S	AGC	S	AGT	K	AAG		
HA1	54	H	CAC	H	CAC	H	CAT		
HA1	55	N	AAC	N	AAT	N	AAC		Cand1(All)
HA1	56	G	GGA	G	GGA	G	GGG		
HA1	57	K	AAA	K	AAA	K	AAA		
HA1	58	L	CTA	L	CTA	L	CTA		
HA1 globolar head	59	C	TGT	C	TGT	C	TGC		
HA1 globolar head	60	K	AAA	L	CTA	K	AAA		
HA1 globolar head	61	L	TTA	L	TTA	L	CTA		
HA1 globolar head	62	K	AAA	K	AAA	R	AGA		
HA1 globolar head	63	G	GGA	G	GGA	G	GGG		
HA1 globolar head	64	I	ATA	I	ATA	V	GTA		
HA1 globolar head	65	A	GCC	A	GCC	A	GCC		
HA1 globolar head	66	P	CCA	P	CCA	P	CCA		
HA1 globolar head	67	L	TTA	L	CTA	L	TTG		
HA1 globolar head	68	Q	CAA	Q	CAA	H	CAT		
HA1 globolar head	69	L	TTG	L	TTG	L	TTG		
HA1 globolar head	70	G	GGG	G	GGT	G	GGT		
HA1 globolar head	71	K	AAA	N	AAT	K	AAA		sequon(BR2007)
HA1 globolar head	72	C	TGT	C	TGC	C	TGT		
HA1 globolar head	73	N	AAT	S	AGC	N	AAC		Cand1(SC1918), Cand1(CA2009)
HA1 globolar head	74	I	ATC	V	GTT	I	ATT		
HA1 globolar head	75	A	GCC	A	GCC	A	GCT		
HA1 globolar head	76	G	GGA	G	GGG	G	GGC		
HA1 globolar head	77	W	TGG	W	TGG	W	TGG		
HA1 globolar head	78	L	CTC	I	ATC	I	ATC		
HA1 globolar head	79	L	TTG	L	TTA	L	CTG		
HA1 globolar head	80	G	GGA	G	GGA	G	GGA		
HA1 globolar head	81	N	AAC	N	AAC	N	AAT		
HA1 globolar head	82	P	CCG	P	CCA	P	CCA		
HA1 globolar head	83	E	GAA	E	GAA	E	GAG		
HA1 globolar head	84	C	TGC	C	TGC	C	TGT		
HA1 globolar head	85	D	GAT	E	GAA	E	GAA		
HA1 globolar head	86	L	TTA	L	TTA	S	TCA		
HA1 globolar head	87	L	CTG	L	CTG	L	CTC	Cb	
HA1 globolar head	88	L	CTC	I	ATT	S	TCC	Cb	
HA1 globolar head	89	T	ACA	S	TCC	T	ACA	Cb	
HA1 globolar head	90	A	GCG	K	AAG	A	GCA	Cb	Cand1(BR2007)
HA1 globolar head	91	S	AGC	E	GAG	S	AGC	Cb	
HA1 globolar head	92	S	TCA	S	TCA	S	TCA	Cb	
HA1 globolar head	93	W	TGG	W	TGG	W	TGG		

HA1 globolar head	94	S	TCC	S	TCC	S	TCC		
HA1 globolar head	95	Y	TAT	Y	TAC	Y	TAC		
HA1 globolar head	96	I	ATT	I	ATT	I	ATT		
HA1 globolar head	97	V	GTA	V	GTA	V	GTG		
HA1 globolar head	98	E	GAA	E	GAA	E	GAA		
HA1 globolar head	99	T	ACA	K	AAA	T	ACA		
HA1 globolar head	100	S	TCG	P	CCA	P	CCT		
HA1 globolar head	101	N	AAC	N	AAT	S	AGT		
HA1 globolar head	102	S	TCA	P	CCT	S	TCA		
HA1 globolar head	103	E	GAG	E	GAG	D	GAC		
HA1 globolar head	104	N	AAT	N	AAT	N	AAT		sequon(All)
HA1 globolar head	105	G	GGA	G	GGA	G	GGA		
HA1 globolar head	106	T	ACA	T	ACA	T	ACG		
HA1 globolar head	107	C	TGT	C	TGT	C	TGT		
HA1 globolar head	108	Y	TAC	Y	TAC	Y	TAC		
HA1 globolar head	109	P	CCA	P	CCA	P	CCA		
HA1 globolar head	110	G	GGA	G	GGG	G	GGA		
HA1 globolar head	111	D	GAT	H	CAT	D	GAT		
HA1 globolar head	112	F	TTC	F	TTC	F	TTC		
HA1 globolar head	113	I	ATC	A	GCT	I	ATC		
HA1 globolar head	114	D	GAC	D	GAC	D	GAT		
HA1 globolar head	115	Y	TAT	Y	TAT	Y	TAT		
HA1 globolar head	116	E	GAA	E	GAG	E	GAG		
HA1 globolar head	117	E	GAA	E	GAA	E	GAG		
HA1 globolar head	118	L	CTG	L	CTG	L	CTA		
HA1 globolar head	119	R	AGG	R	AGG	R	AGA		
HA1 globolar head	120	E	GAG	E	GAG	E	GAG		
HA1 globolar head	121	Q	CAA	Q	CAA	Q	CAA		
HA1 globolar head	122	L	TTG	L	TTG	L	TTG		
HA1 globolar head	123	S	AGC	S	AGT	S	AGC		
HA1 globolar head	124	S	TCA	S	TCA	S	TCA		
HA1 globolar head	125	V	GTG	V	GTA	V	GTG		
HA1 globolar head	126	S	TCA	S	TCT	S	TCA		
HA1 globolar head	127	S	TCG	S	TCA	S	TCA		
HA1 globolar head	128	F	TTT	F	TTT	F	TTT		
HA1 globolar head	129	E	GAA	E	GAG	E	GAA		
HA1 globolar head	130	K	AAA	R	AGG	R	AGG		
HA1 globolar head	131	F	TTC	F	TTC	F	TTT		
HA1 globolar head	132	E	GAA	E	GAA	E	GAG		
HA1 globolar head	133	I	ATA	I	ATA	I	ATA		
HA1 globolar head	134	F	TTT	F	TTC	F	TTC		
HA1 globolar head	135	P	CCC	P	CCC	P	CCC		
HA1 globolar head	136	K	AAG	K	AAA	K	AAG		Cand1(All)
HA1 globolar head	137	T	ACA	E	GAA	T	ACA		
HA1 globolar head	138	S	AGC	S	AGC	S	AGT		
HA1 globolar head	139	S	TCG	S	TCA	S	TCA		
HA1 globolar head	140	W	TGG	W	TGG	W	TGG		
HA1 globolar head	141	P	CCC	P	CCC	P	CCC	Sa	
HA1 globolar head	142	N	AAT	N	AAC	N	AAT	Sa	sequon(BR2007)
HA1 globolar head	143	H	CAT	H	CAC	H	CAT		Cand1(SC1918), Cand1(CA2009)
HA1 globolar head	144	E	GAA	T	ACC	D	GAC		Cand1(BR2007)
HA1 globolar head	145	T	ACA	V	GTA	S	TCG		
HA1 globolar head	146	T	ACC	T	ACC	N	AAC		Cand1(CA2009)
HA1 globolar head	147	K	AAA	－	－	K	AAA		
HA1 globolar head	148	G	GGT	G	GGA	G	GGT		
HA1 globolar head	149	V	GTA	V	GTG	V	GTA		
HA1 globolar head	150	T	ACG	S	TCA	T	ACG		
HA1 globolar head	151	A	GCA	A	GCA	A	GCA		
HA1 globolar head	152	A	GCA	S	TCA	A	GCA		
HA1 globolar head	153	C	TGC	C	TGC	C	TGT		
HA1 globolar head	154	S	TCC	S	TCC	P	CCT	Ca	
HA1 globolar head	155	Y	TAT	H	CAT	H	CAT	Ca	
HA1 globolar head	156	A	GCG	N	AAT	A	GCT	Ca	
HA1 globolar head	157	G	GGA	G	GGG	G	GGA	Ca	
HA1 globolar head	158	A	GCA	E	GAA	A	GCA	Ca	
HA1 globolar head	159	S	AGC	S	AGC	K	AAA	Ca	
HA1 globolar head	160	S	AGT	S	AGT	S	AGC		
HA1 globolar head	161	F	TTT	F	TTT	F	TTC		
HA1 globolar head	162	Y	TAC	Y	TAC	Y	TAC		
HA1 globolar head	163	R	AGA	R	AGA	K	AAA		
HA1 globolar head	164	N	AAT	N	AAT	N	AAT		Cand1(CA2009)
HA1 globolar head	165	L	TTG	L	TTG	L	TTA		
HA1 globolar head	166	L	CTG	L	CTA	I	ATA		
HA1 globolar head	167	W	TGG	W	TGG	W	TGG		
HA1 globolar head	168	L	CTG	L	CTG	L	CTA		
HA1 globolar head	169	T	ACA	T	ACG	V	GTT		
HA1 globolar head	170	K	AAG	G	GGG	K	AAA	Sa	
HA1 globolar head	171	K	AAG	K	AAG	K	AAA	Sa	Cand1(SC1918)
HA1 globolar head	172	G	GGA	N	AAT	G	GGA	Sa	Cand1(BR2007)
HA1 globolar head	173	S	AGC	G	GGT	N	AAT	Sa	Cand1(CA2009)
HA1 globolar head	174	S	TCA	L	TTG	S	TCA	Sa	
HA1 globolar head	175	Y	TAC	Y	TAC	Y	TAC		
HA1 globolar head	176	P	CCA	P	CCA	P	CCA	Sa	
HA1 globolar head	177	K	AAG	N	AAC	K	AAG	Sa	sequon(BR2007), Cand1(SC1918), Cand1(CA2009)
HA1 globolar head	178	L	CTT	L	CTG	L	CTC	Sa	
HA1 globolar head	179	S	AGC	S	AGC	S	AGC	Sa	Cand1(All)
HA1 globolar head	180	K	AAG	K	AAG	K	AAA	Sa	
HA1 globolar head	181	S	TCC	S	TCC	S	TCC	Sa	
HA1 globolar head	182	Y	TAT	Y	TAT	Y	TAC		
HA1 globolar head	183	V	GTG	A	GCA	I	ATT	Ca	
HA1 globolar head	184	N	AAC	N	AAC	N	AAT	Ca	Cand1(All)
HA1 globolar head	185	N	AAT	N	AAC	D	GAT	Ca	
HA1 globolar head	186	K	AAA	K	AAA	K	AAA	Ca	
HA1 globolar head	187	G	GGG	E	GAA	G	GGG	Ca	
HA1 globolar head	188	K	AAA	K	AAA	K	AAA		
HA1 globolar head	189	E	GAA	E	GAA	E	GAA		
HA1 globolar head	190	V	GTC	V	GTC	V	GTC		

HA1 globolar head	191	L	CTT	L	CTT	L	CTC		
HA1 globolar head	192	V	GTA	V	GTA	V	GTG		
HA1 globolar head	193	L	CTA	L	CTA	L	CTA		
HA1 globolar head	194	W	TGG	W	TGG	W	TGG		
HA1 globolar head	195	G	GGT	G	GGT	G	GGC		
HA1 globolar head	196	V	GTT	V	GTT	I	ATT		
HA1 globolar head	197	H	CAT	H	CAT	H	CAC		
HA1 globolar head	198	H	CAT	H	CAC	H	CAT		
HA1 globolar head	199	P	CCG	P	CCG	P	CCA		
HA1 globolar head	200	P	CCT	P	CCA	S	TCT		
HA1 globolar head	201	T	ACC	N	AAC	T	ACT	Sb	Cand1(SC1918), Cand1(BR2007)
HA1 globolar head	202	G	GGT	I	ATA	S	AGT	Sb	
HA1 globolar head	203	T	ACT	G	GGT	A	GCT	Sb	
HA1 globolar head	204	D	GAT	D	GAC	D	GAC	Sb	
HA1 globolar head	205	Q	CAA	Q	CAA	Q	CAA	Sb	
HA1 globolar head	206	Q	CAG	K	AAG	Q	CAA	Sb	
HA1 globolar head	207	S	AGT	A	GCC	S	AGT	Sb	
HA1 globolar head	208	L	CTC	L	CTC	L	CTC	Sb	
HA1 globolar head	209	Y	TAT	Y	TAT	Y	TAT	Sb	Cand1(BR2007)
HA1 globolar head	210	Q	CAG	H	CAT	Q	CAG	Sb	
HA1 globolar head	211	N	AAT	T	ACA	N	AAT	Sb	
HA1 globolar head	212	A	GCA	E	GAA	A	GCA	Sb	
HA1 globolar head	213	D	GAT	N	AAT	D	GAT		Cand1(BR2007)
HA1 globolar head	214	A	GCT	A	GCT	T	ACA		
HA1 globolar head	215	Y	TAT	Y	TAT	Y	TAT		Cand1(SC1918), Cand1(BR2007)
HA1 globolar head	216	V	GTC	V	GTC	V	GTT		
HA1 globolar head	217	S	TCT	S	TCT	F	TTT		
HA1 globolar head	218	V	GTA	V	GTA	V	GTG		
HA1 globolar head	219	G	GGG	V	GTG	G	GGG		
HA1 globolar head	220	S	TCA	S	TCT	S	TCA	Ca	
HA1 globolar head	221	S	TCA	S	TCA	S	TCA	Ca	
HA1 globolar head	222	K	AAA	H	CAT	R	AGA	Ca	Cand1(BR2007)
HA1 globolar head	223	Y	TAT	Y	TAT	Y	TAC		
HA1 globolar head	224	N	AAC	S	AGC	S	AGC		Cand1(SC1918)
HA1 globolar head	225	R	AGG	R	AGA	K	AAG		
HA1 globolar head	226	R	AGA	K	AAA	K	AAG		Cand1(BR2007)
HA1 globolar head	227	F	TTC	F	TTC	F	TTC		
HA1 globolar head	228	T	ACC	T	ACC	K	AAG		
HA1 globolar head	229	P	CCG	P	CCA	P	CCG		
HA1 globolar head	230	E	GAA	E	GAA	E	GAA		
HA1 globolar head	231	I	ATA	I	ATA	I	ATA		
HA1 globolar head	232	A	GCA	A	GCC	A	GCA		
HA1 globolar head	233	A	GCG	K	AAA	I	ATA		
HA1 globolar head	234	R	AGA	R	AGA	R	AGA		
HA1 globolar head	235	P	CCC	P	CCC	P	CCC		
HA1 globolar head	236	K	AAA	K	AAA	K	AAA		
HA1 globolar head	237	V	GTA	V	GTA	V	GTG		
HA1 globolar head	238	R	AGA	R	AGA	R	AGG	Ca	
HA1 globolar head	239	D	GAT	D	GAT	D	GAT	Ca	
HA1 globolar head	240	Q	CAA	Q	CAA	Q	CAA		
HA1 globolar head	241	A	GCT	E	GAA	E	GAA		
HA1 globolar head	242	G	GGG	G	GGA	G	GGG		
HA1 globolar head	243	R	AGG	R	AGA	R	AGA		
HA1 globolar head	244	M	ATG	I	ATC	M	ATG		
HA1 globolar head	245	N	AAC	N	AAT	N	AAC		Cand1(All)
HA1 globolar head	246	Y	TAT	Y	TAC	Y	TAT		
HA1 globolar head	247	Y	TAC	Y	TAC	Y	TAC		Cand1(All)
HA1 globolar head	248	W	TGG	W	TGG	W	TGG		
HA1 globolar head	249	T	ACA	T	ACT	T	ACA		
HA1 globolar head	250	L	TTA	L	CTG	L	CTA		
HA1 globolar head	251	L	CTA	L	CTT	V	GTA		
HA1 globolar head	252	E	GAA	E	GAA	E	GAG	Ca	
HA1 globolar head	253	P	CCC	P	CCC	P	CCG	Ca	
HA1 globolar head	254	G	GGA	G	GGG	G	GGA	Ca	
HA1 globolar head	255	D	GAC	D	GAT	D	GAC		
HA1 globolar head	256	T	ACA	T	ACA	K	AAA		Cand1(CA2009)
HA1 globolar head	257	I	ATA	I	ATA	I	ATA		
HA1 globolar head	258	T	ACA	I	ATA	T	ACA		
HA1 globolar head	259	F	TTT	F	TTT	F	TTC		
HA1 globolar head	260	E	GAG	E	GAG	E	GAA		
HA1 globolar head	261	A	GCA	A	GCA	A	GCA		
HA1 globolar head	262	T	ACT	N	AAT	T	ACT		Cand1(BR2007)
HA1 globolar head	263	G	GGA	G	GGA	G	GGA		
HA1 globolar head	264	N	AAT	N	AAT	N	AAT		Cand1(SC1918), Cand1(BR2007)
HA1 globolar head	265	L	CTA	L	CTA	L	CTA		
HA1 globolar head	266	I	ATA	I	ATA	V	GTG		
HA1 globolar head	267	A	GCA	A	GCG	V	GTA		
HA1 globolar head	268	P	CCA	P	CCA	P	CCG		
HA1 globolar head	269	W	TGG	R	AGA	R	AGA		
HA1 globolar head	270	Y	TAT	Y	TAT	Y	TAT		
HA1 globolar head	271	A	GCT	A	GCT	A	GCA		
HA1 globolar head	272	F	TTC	F	TTC	F	TTC		
HA1 globolar head	273	A	GCA	A	GCA	A	GCA		
HA1 globolar head	274	L	CTG	L	CTG	M	ATG		
HA1 globolar head	275	N	AAT	S	AGT	E	GAA		Cand1(SC1918)
HA1 globolar head	276	R	AGA	R	AGA	R	AGA		
HA1 globolar head	277	G	GGT	G	GGC	N	AAT		
HA1 globolar head	278	S	TCT	F	TTT	A	GCT		
HA1 globolar head	279	G	GGA	G	GGA	G	GGA		
HA1 globolar head	280	S	TCC	S	TCA	S	TCT		
HA1 globolar head	281	G	GGT	G	GGA	G	GGT		
HA1 globolar head	282	I	ATC	I	ATC	I	ATT		Cand1(SC1918)
HA1 globolar head	283	I	ATC	I	ATC	I	ATC		Cand1(All)
HA1 globolar head	284	T	ACT	N	AAC	I	ATT		Cand1(BR2007)
HA1 globolar head	285	S	TCA	S	TCA	S	TCA		
HA1 globolar head	286	D	GAC	N	AAT	D	GAT		Cand1(BR2007)
HA1 globolar head	287	A	GCA	A	GCA	T	ACA		

HA1 globolar head	288	P	CCA	P	CCA	P	CCA		
HA1 globolar head	289	V	GTG	M	ATG	V	GTC		
HA1 globolar head	290	H	CAT	D	GAT	H	CAC		
HA1 globolar head	291	D	GAT	K	AAA	D	GAT		
HA1 globolar head	292	C	TGT	C	TGT	C	TGC		
HA1	293	N	AAC	D	GAT	N	AAT		sequon(CA2009), Cand1(SC1918)
HA1	294	T	ACG	A	GCG	T	ACA		
HA1	295	K	AAG	K	AAG	T	ACT		
HA1	296	C	TGT	C	TGC	C	TGT		
HA1	297	Q	CAA	Q	CAA	Q	CAA		
HA1	298	T	ACA	T	ACA	T	ACA		
HA1	299	P	CCC	P	CCT	P	CCC		
HA1	300	H	CAT	Q	CAG	K	AAG		
HA1	301	G	GGT	G	GGA	G	GGT		
HA1	302	A	GCT	A	GCT	A	GCT		
HA1	303	I	ATA	I	ATA	I	ATA		
HA1	304	N	AAC	N	AAC	N	AAC		sequon(All)
HA1	305	S	AGC	S	AGC	T	ACC		
HA1	306	S	AGT	S	AGT	S	AGC		
HA1	307	L	CTC	L	CTT	L	CTC		
HA1	308	P	CCT	P	CCT	P	CCA		
HA1	309	F	TTC	F	TTC	F	TTT		
HA1	310	Q	CAG	Q	CAG	Q	CAG		
HA1	311	N	AAT	N	AAC	N	AAT		
HA1	312	I	ATA	V	GTA	I	ATA		
HA1	313	H	CAT	H	CAC	H	CAT		
HA1	314	P	CCA	P	CCA	P	CCG		
HA1	315	V	GTC	V	GTC	I	ATC		
HA1	316	T	ACA	T	ACA	T	ACA		
HA1	317	I	ATA	I	ATA	I	ATT		
HA1	318	G	GGA	G	GGA	G	GGA		
HA1	319	E	GAG	E	GAG	K	AAA		
HA1	320	C	TGC	C	TGT	C	TGT		
HA1	321	P	CCA	P	CCA	P	CCA		
HA1	322	K	AAA	K	AAG	K	AAA		
HA1	323	Y	TAC	Y	TAT	Y	TAT		
HA1	324	V	GTC	V	GTC	V	GTA		
HA1	325	R	AGG	R	AGG	K	AAA		Cand1(CA2009)
HA1	326	S	AGT	S	AGT	S	AGC		
HA1	327	T	ACC	A	GCA	T	ACA		
HA1	328	K	AAA	K	AAA	K	AAA		
HA1	329	L	TTG	L	TTA	L	TTG		
HA1	330	R	AGG	R	AGG	R	AGA		
HA1	331	M	ATG	M	ATG	L	CTG		
HA1	332	A	GCT	V	GTT	A	GCC		
HA1	333	T	ACA	T	ACA	T	ACA		
HA1	334	G	GGA	G	GGA	G	GGA		
HA1	335	L	CTA	L	CTA	L	TTG		
HA1	336	R	AGA	R	AGG	R	AGG		
HA1	337	N	AAC	N	AAC	N	AAT		Cand1(All)
HA1	338	I	ATT	I	ATC	I	ATC		
HA1	339	P	CCA	P	CCA	P	CCG		
HA1	340	S	TCT	S	TCC	S	TCT		
HA1	341	I	ATT	I	ATT	I	ATT		Cand1(All)
HA1	342	Q	CAA	Q	CAA	Q	CAA		
HA1	343	S	TCC	S	TCC	S	TCT		
Cleavage site	344	R	AGG	R	AGA	R	AGA		
HA2	345	G	GGT	G	GGT	G	GGC		
HA2	346	L	CTA	L	TTG	L	CTA		
HA2	347	F	TTT	F	TTT	F	TTT		
HA2	348	G	GGA	G	GGA	G	GGG		
HA2	349	A	GCC	A	GCC	A	GCC		
HA2	350	I	ATT	I	ATT	I	ATT		
HA2	351	A	GCC	A	GCC	A	GCC		
HA2	352	G	GGT	G	GGT	G	GGT		
HA2	353	F	TTT	F	TTC	F	TTC		
HA2	354	I	ATT	I	ATT	I	ATT		
HA2	355	E	GAG	E	GAA	E	GAA		
HA2	356	G	GGG	G	GGG	G	GGG		
HA2	357	G	GGA	G	GGG	G	GGG		
HA2	358	W	TGG	W	TGG	W	TGG		
HA2	359	T	ACT	T	ACT	T	ACA		
HA2	360	G	GGA	G	GGA	G	GGG		
HA2	361	M	ATG	M	ATG	M	ATG		
HA2	362	I	ATA	V	GTA	V	GTA		
HA2	363	D	GAT	D	GAT	D	GAT		
HA2	364	G	GGA	G	GGT	G	GGA		
HA2	365	W	TGG	W	TGG	W	TGG		
HA2	366	Y	TAT	Y	TAT	Y	TAC		
HA2	367	G	GGT	G	GGT	G	GGT		
HA2	368	Y	TAT	Y	TAT	Y	TAT		
HA2	369	H	CAT	H	CAT	H	CAC		
HA2	370	H	CAT	H	CAT	H	CAT		
HA2	371	Q	CAG	Q	CAG	Q	CAA		
HA2	372	N	AAT	N	AAT	N	AAT		
HA2	373	E	GAA	E	GAG	E	GAG		
HA2	374	Q	CAG	Q	CAA	Q	CAG		
HA2	375	G	GGA	G	GGA	G	GGG		
HA2	376	S	TCA	S	TCT	S	TCA		
HA2	377	G	GGC	G	GGC	G	GGA		
HA2	378	Y	TAT	Y	TAT	Y	TAT		
HA2	379	A	GCA	A	GCT	A	GCA		
HA2	380	A	GCG	A	GCA	A	GCC		
HA2	381	D	GAT	D	GAT	D	GAC		
HA2	382	Q	CAA	Q	CAA	L	CTG		
HA2	383	K	AAA	K	AAA	K	AAG		Cand1(All)
HA2	384	S	AGC	S	AGC	S	AGC		

HA2	385	T	ACA	T	ACA	T	ACA		
HA2	386	Q	CAA	Q	CAA	Q	CAG		
HA2	387	N	AAT	N	AAT	N	AAT		Cand1(All)
HA2	388	A	GCC	A	GCC	A	GCC		
HA2	389	I	ATT	I	ATT	I	ATT		
HA2	390	D	GAC	N	AAT	D	GAC		Cand1(BR2007)
HA2	391	G	GGG	G	GGG	E	GAG		
HA2	392	I	ATT	I	ATT	I	ATT		
HA2	393	T	ACA	T	ACA	T	ACT		
HA2	394	N	AAC	N	AAC	N	AAC		
HA2	395	K	AAG	K	AAG	K	AAA		
HA2	396	V	GTG	V	GTG	V	GTA		
HA2	397	N	AAT	N	AAT	N	AAT		
HA2	398	S	TCT	S	TCT	S	TCT		
HA2	399	V	GTT	V	GTA	V	GTT		
HA2	400	I	ATC	I	ATT	I	ATT		
HA2	401	E	GAG	E	GAG	E	GAA		
HA2	402	K	AAA	K	AAA	K	AAG		
HA2	403	M	ATG	M	ATG	M	ATG		
HA2	404	N	AAC	N	AAC	N	AAT		
HA2	405	T	ACC	T	ACT	T	ACA		
HA2	406	Q	CAA	Q	CAA	Q	CAG		
HA2	407	F	TTC	F	TTC	F	TTC		
HA2	408	T	ACA	T	ACA	T	ACA		
HA2	409	A	GCA	A	GCA	A	GCA		
HA2	410	V	GTG	V	GTG	V	GTA		
HA2	411	G	GGT	G	GGC	G	GGT		
HA2	412	K	AAA	K	AAA	K	AAA		
HA2	413	E	GAA	E	GAA	E	GAG		
HA2	414	F	TTC	F	TTC	F	TTC		
HA2	415	N	AAC	N	AAC	N	AAC		Cand1(SC1918), Cand1(BR2007)
HA2	416	N	AAC	K	AAA	H	CAC		
HA2	417	L	TTA	L	TTG	L	CTG		
HA2	418	E	GAA	E	GAA	E	GAA		
HA2	419	R	AGA	R	AGA	K	AAA		
HA2	420	R	AGG	R	AGG	R	AGA		
HA2	421	I	ATA	M	ATG	I	ATA		
HA2	422	E	GAA	E	GAA	E	GAG		
HA2	423	N	AAT	N	AAC	N	AAT		Cand1(All)
HA2	424	L	TTA	L	TTG	L	TTA		
HA2	425	N	AAT	N	AAT	N	AAT		Cand1(All)
HA2	426	K	AAA	K	AAA	K	AAA		
HA2	427	K	AAA	K	AAA	K	AAA		
HA2	428	V	GTC	V	GTT	V	GTT		
HA2	429	D	GAT	D	GAT	D	GAT		
HA2	430	D	GAT	D	GAT	D	GAT		
HA2	431	G	GGA	G	GGG	G	GGT		
HA2	432	F	TTT	F	TTT	F	TTC		
HA2	433	L	CTG	I	ATA	L	CTG		
HA2	434	D	GAT	D	GAC	D	GAC		
HA2	435	I	ATT	I	ATT	I	ATT		Cand1(All)
HA2	436	W	TGG	W	TGG	W	TGG		
HA2	437	T	ACA	T	ACA	T	ACT		
HA2	438	Y	TAT	Y	TAT	Y	TAC		
HA2	439	N	AAT	N	AAT	N	AAT		
HA2	440	A	GCA	A	GCA	A	GCC		
HA2	441	E	GAA	E	GAA	E	GAA		
HA2	442	L	TTG	L	CTG	L	CTG		
HA2	443	L	TTA	L	TTG	L	TTG		
HA2	444	V	GTT	V	GTT	V	GTT		
HA2	445	L	CTA	L	CTA	L	CTA		
HA2	446	L	CTG	L	CTG	L	TTG		
HA2	447	E	GAA	E	GAA	E	GAA		
HA2	448	N	AAT	N	AAT	N	AAT		Cand1(All)
HA2	449	E	GAA	E	GAA	E	GAA		
HA2	450	R	AGA	R	AGG	R	AGA		
HA2	451	T	ACC	T	ACT	T	ACT		
HA2	452	L	CTG	L	TTG	L	TTG		
HA2	453	D	GAT	D	GAT	D	GAC		
HA2	454	F	TTC	F	TTC	Y	TAC		
HA2	455	H	CAT	H	CAT	H	CAC		Cand1(All)
HA2	456	D	GAC	D	GAC	D	GAT		
HA2	457	S	TCA	S	TCC	S	TCA		
HA2	458	N	AAT	N	AAT	N	AAT		Cand1(All)
HA2	459	V	GTA	V	GTG	V	GTG		
HA2	460	R	AGG	K	AAG	K	AAG		
HA2	461	N	AAT	N	AAT	N	AAC		Cand1(All)
HA2	462	L	CTG	L	CTG	L	TTA		
HA2	463	Y	TAT	Y	TAT	Y	TAT		
HA2	464	E	GAG	E	GAG	E	GAA		
HA2	465	K	AAA	K	AAA	K	AAG		
HA2	466	V	GTA	V	GTA	V	GTA		
HA2	467	K	AAA	K	AAA	R	AGA		
HA2	468	S	AGC	S	AGC	S	AGC		
HA2	469	Q	CAA	Q	CAG	Q	CAG		
HA2	470	L	TTA	L	TTA	L	CTA		
HA2	471	K	AAG	K	AAG	K	AAA		
HA2	472	N	AAT	N	AAT	N	AAC		Cand1(All)
HA2	473	N	AAT	N	AAT	N	AAT		Cand1(All)
HA2	474	A	GCC	A	GCT	A	GCC		
HA2	475	K	AAG	K	AAA	K	AAG		
HA2	476	E	GAA	E	GAA	E	GAA		
HA2	477	I	ATC	I	ATA	I	ATT		
HA2	478	G	GGA	G	GGA	G	GGA		
HA2	479	N	AAT	N	AAT	N	AAC		Cand1(All)
HA2	480	G	GGA	G	GGG	G	GGC		
HA2	481	C	TGT	C	TGT	C	TGC		

HA2	482	F	TTT	F	TTT	F	TTT		
HA2	483	E	GAG	E	GAA	E	GAA		
HA2	484	F	TTC	F	TTC	F	TTT		
HA2	485	Y	TAC	Y	TAT	Y	TAC		
HA2	486	H	CAC	H	CAC	H	CAC		
HA2	487	K	AAG	K	AAG	K	AAA		
HA2	488	C	TGT	C	TGT	C	TGC		
HA2	489	D	GAC	N	AAC	D	GAT		Cand1(CA2009)
HA2	490	D	GAT	D	GAT	N	AAC		Cand1(CA2009)
HA2	491	A	GCA	E	GAA	T	ACG		
HA2	492	C	TGC	C	TGC	C	TGC		
HA2	493	M	ATG	M	ATG	M	ATG		
HA2	494	E	GAA	E	GAG	E	GAA		
HA2	495	S	AGT	S	AGT	S	AGT		
HA2	496	V	GTA	V	GTA	V	GTC		
HA2	497	R	AGA	K	AAG	K	AAA		
HA2	498	N	AAT	N	AAT	N	AAT		sequon(All)
HA2	499	G	GGG	G	GGA	G	GGG		
HA2	500	T	ACT	T	ACT	T	ACT		
HA2	501	Y	TAT	Y	TAT	Y	TAT		
HA2	502	D	GAT	D	GAC	D	GAC		
HA2	503	Y	TAC	Y	TAT	Y	TAC		
HA2	504	P	CCA	P	CCA	P	CCA		
HA2	505	K	AAA	K	AAA	K	AAA		Cand1(All)
HA2	506	Y	TAT	Y	TAT	Y	TAC		
HA2	507	S	TCA	S	TCC	S	TCA		
HA2	508	E	GAA	E	GAA	E	GAG		
HA2	509	E	GAA	E	GAA	E	GAA		
HA2	510	S	TCA	S	TCA	A	GCA		
HA2	511	K	AAG	K	AAG	K	AAA		
HA2	512	L	TTG	L	TTA	L	TTA		
HA2	513	N	AAC	N	AAC	N	AAC		
HA2	514	R	AGA	R	AGG	R	AGA		
HA2	515	E	GAA	E	GAG	E	GAA		
HA2	516	E	GAA	K	AAA	E	GAA		
HA2	517	I	ATA	I	ATT	I	ATA		
HA2	518	D	GAT	D	GAT	D	GAT		
HA2	519	G	GGA	G	GGA	G	GGG		
HA2	520	V	GTG	V	GTG	V	GTA		
HA2	521	K	AAA	K	AAA	K	AAG		
HA2	522	L	TTA	L	TTG	L	CTG		
HA2	523	E	GAA	E	GAA	E	GAA		
HA2	524	S	TCA	S	TCA	S	TCA		
HA2	525	M	ATG	M	ATG	T	ACA		
HA2	526	G	GGG	G	GGA	R	AGG		
HA2	527	V	GTC	V	GTC	I	ATT		
HA2	528	Y	TAT	Y	TAT	Y	TAC		
HA2	529	Q	CAG	Q	CAG	Q	CAG		
HA2	530	I	ATT	I	ATT	I	ATT		
HA2	531	L	CTG	L	CTG	L	TTG		
HA2	532	A	GCG	A	GCG	A	GCG		
HA2	533	I	ATC	I	ATC	I	ATC		Cand1(All)
HA2	534	Y	TAC	Y	TAC	Y	TAT		Cand1(All)
HA2	535	S	TCA	S	TCA	S	TCA		
HA2	536	T	ACT	T	ACA	T	ACT		
HA2	537	V	GTC	V	GTC	V	GTC		
HA2	538	A	GCC	A	GCC	A	GCC		
HA2	539	S	AGT	S	AGT	S	AGT		
HA2	540	S	TCA	S	TCT	S	TCA		
HA2	541	L	CTA	L	CTG	L	TTG		
HA2	542	V	GTG	V	GTT	V	GTA		
HA2	543	L	CTG	L	CTT	L	CTG		
HA2	544	L	TTA	L	TTG	V	GTA		
HA2	545	V	GTC	V	GTC	V	GTC		
HA2	546	S	TCC	S	TCC	S	TCC		
HA2	547	L	CTG	L	CTG	L	CTG		
HA2	548	G	GGG	G	GGG	G	GGG		
HA2	549	A	GCA	A	GCA	A	GCA		
HA2	550	I	ATC	I	ATC	I	ATC		
HA2	551	S	AGC	S	AGC	S	AGT		
HA2	552	F	TTC	F	TTC	F	TTC		
HA2	553	W	TGG	W	TGG	W	TGG		
HA2	554	M	ATG	M	ATG	M	ATG		
HA2	555	C	TGT	C	TGT	C	TGC		
HA2	556	S	TCT	S	TCC	S	TCT		
HA2	557	N	AAT	N	AAT	N	AAT		sequon(All)
HA2	558	G	GGG	G	GGG	G	GGG		
HA2	559	S	TCT	S	TCT	S	TCT		
HA2	560	L	TTG	L	TTA	L	CTA		
HA2	561	Q	CAG	Q	CAG	Q	CAG		
HA2	562	C	TGC	C	TGT	C	TGT		
HA2	563	R	AGA	R	AGA	R	AGA		
HA2	564	I	ATA	I	ATA	I	ATA		
HA2	565	C	TGC	C	TGC	C	TGT		
HA2	566	I	ATT	I	ATC	I	ATT		
