# Supplementary material for: Predicting the Antigenic Structure of the Pandemic (H1N1) 2009 Influenza Virus Hemagglutinin
Source: PLoS One. 2010 Jan 1;5(1):e8553. doi: 10.1371/journal.pone.0008553 (PMC2797400; doi:10.1371/journal.pone.0008553)
Supplement: Figure S1 — Amino acid substitutions of seasonal human H1N1 virus HAs shown in close-up views of each antigenic site. The strains used in this analysis are corresponding to those shown in Figure 2. Amino acids are colored according to the scheme in the legend of Figure 1B. (1.02 MB PDF) [file pone.0008553.s002.pdf]

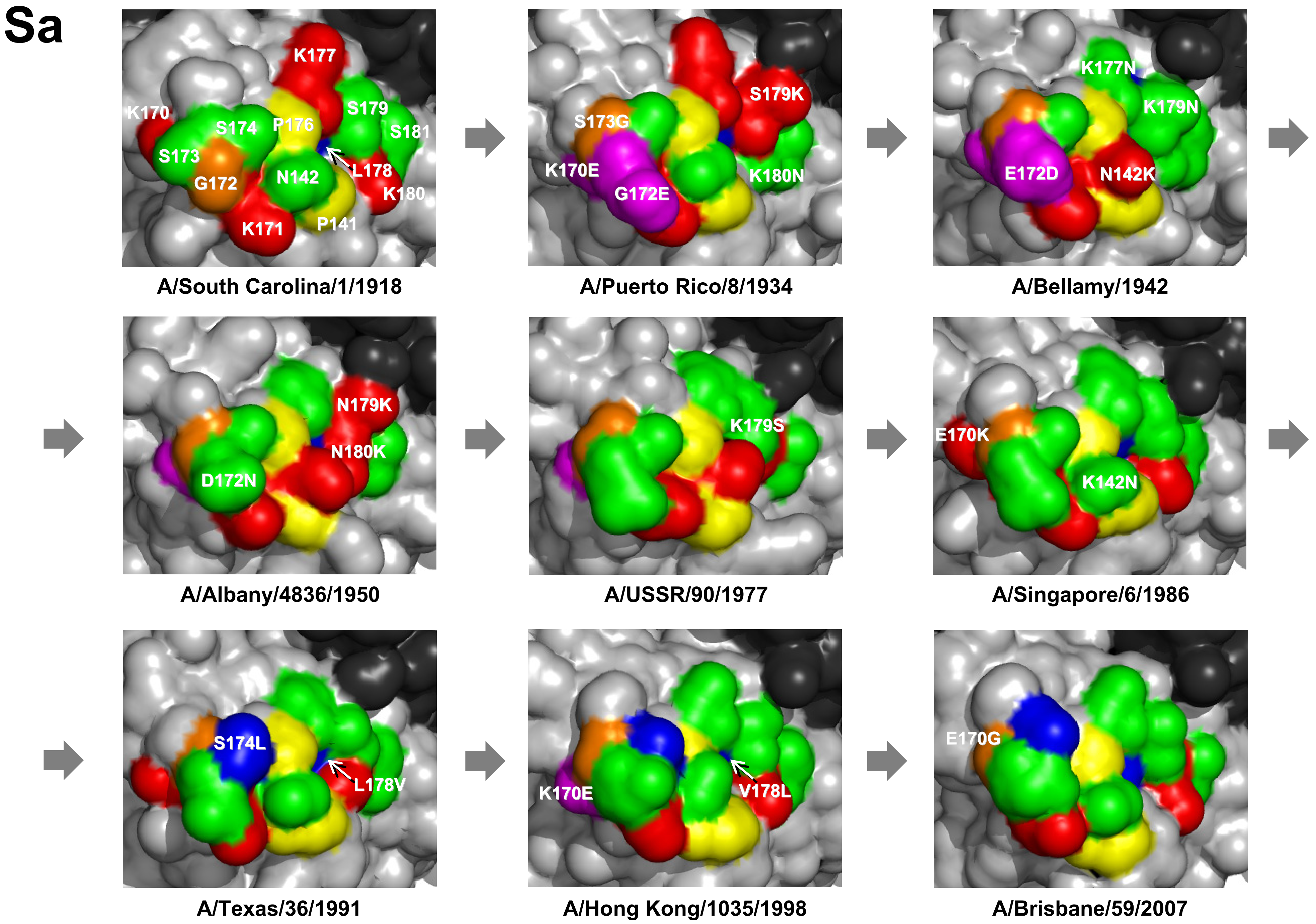
Sa
K177
K170
S174
P176
S179
S181
S173
G172
N142
L178
K180
K171
P141
A/South Carolina/1/1918
S179K
S173G
K170E
G172E
K180N
A/Puerto Rico/8/1934
K177N
K179N
E172D
N142K
A/Bellamy/1942
N179K
N180K
D172N
A/Albany/4836/1950
K179S
A/USSR/90/1977
E170K
K142N
A/Singapore/6/1986
S174L
L178V
A/Texas/36/1991
K170E
V178L
A/Hong Kong/1035/1998
E170G
A/Brisbane/59/2007

Sb

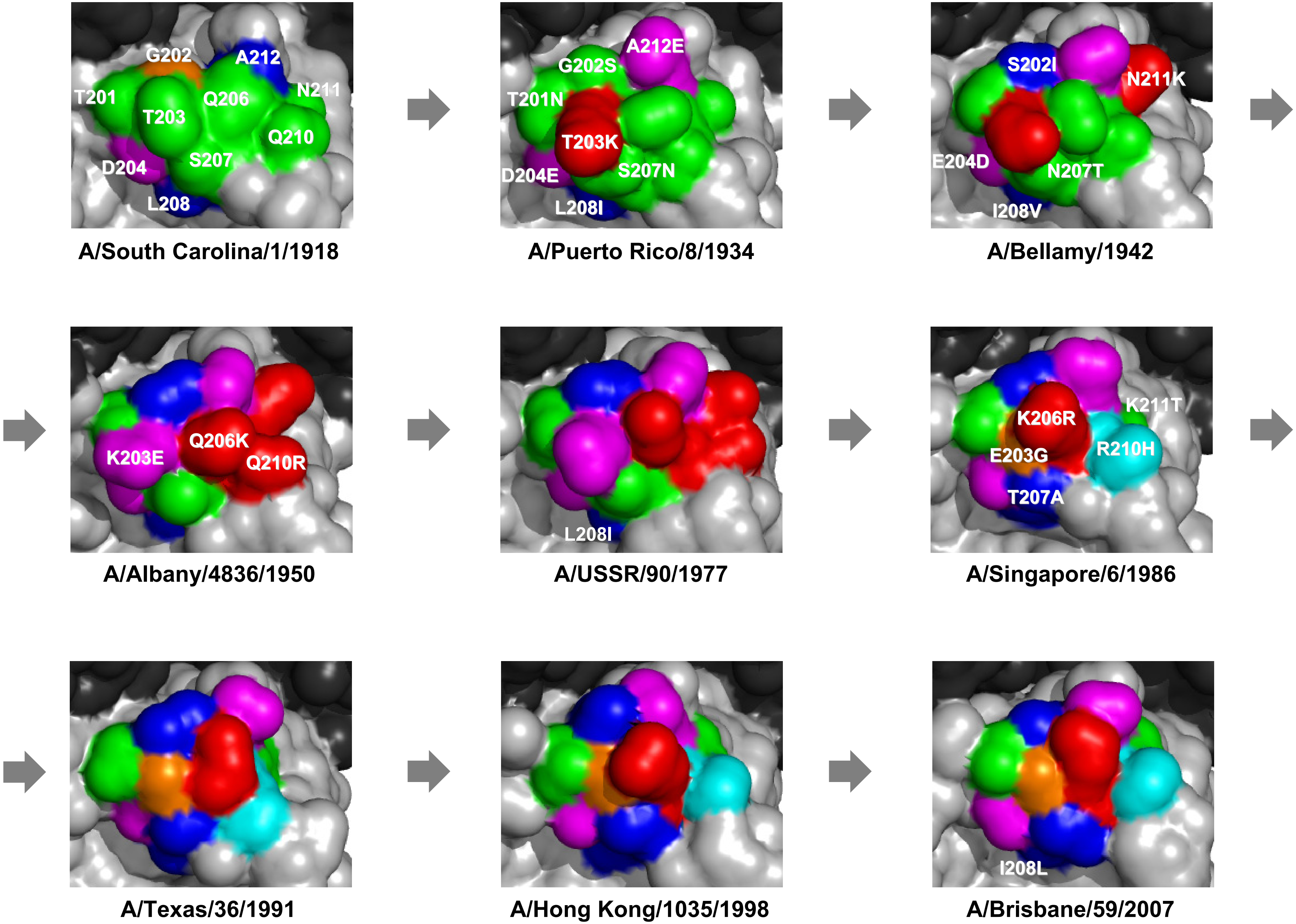
G202
A212
T201
Q206
N211
T203
Q210
D204
S207
L208
A/South Carolina/1/1918
A212E
G202S
T201N
T203K
D204E
S207N
L208I
A/Puerto Rico/8/1934
S202I
N211K
E204D
N207T
I208V
A/Bellamy/1942
Q206K
K203E
Q210R
A/Albany/4836/1950
L208I
A/USSR/90/1977
K211T
K206R
R210H
E203G
T207A
A/Singapore/6/1986
A/Texas/36/1991
A/Hong Kong/1035/1998
I208L
A/Brisbane/59/2007

Ca1

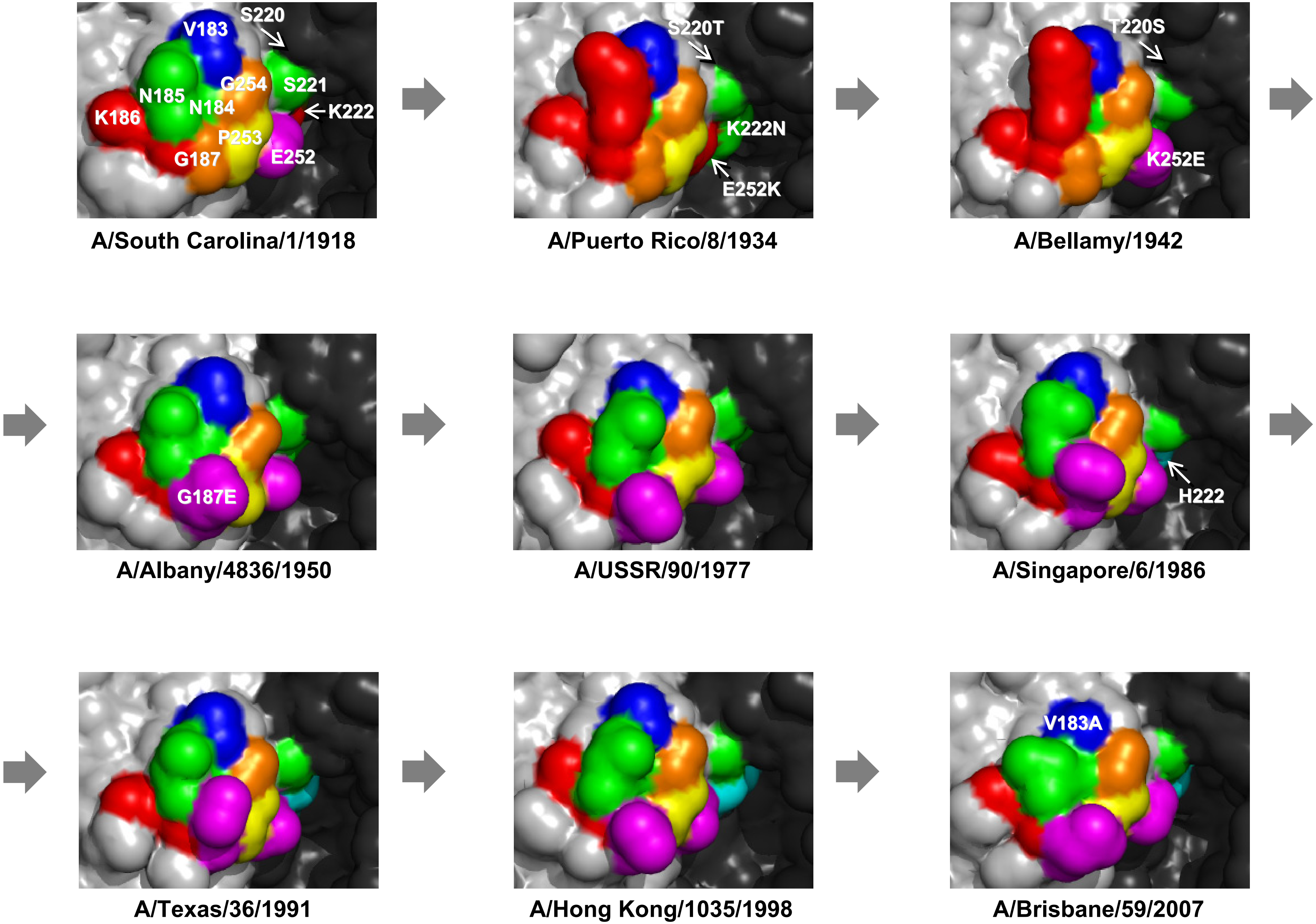
V183
S220
G254
S221
N185
N184
K222
K186
P253
G187
E252
A/South Carolina/1/1918
S220T
K222N
E252K
A/Puerto Rico/8/1934
T220S
K252E
A/Bellamy/1942
G187E
A/Albany/4836/1950
A/USSR/90/1977
H222
A/Singapore/6/1986
A/Texas/36/1991
A/Hong Kong/1035/1998
V183A
A/Brisbane/59/2007

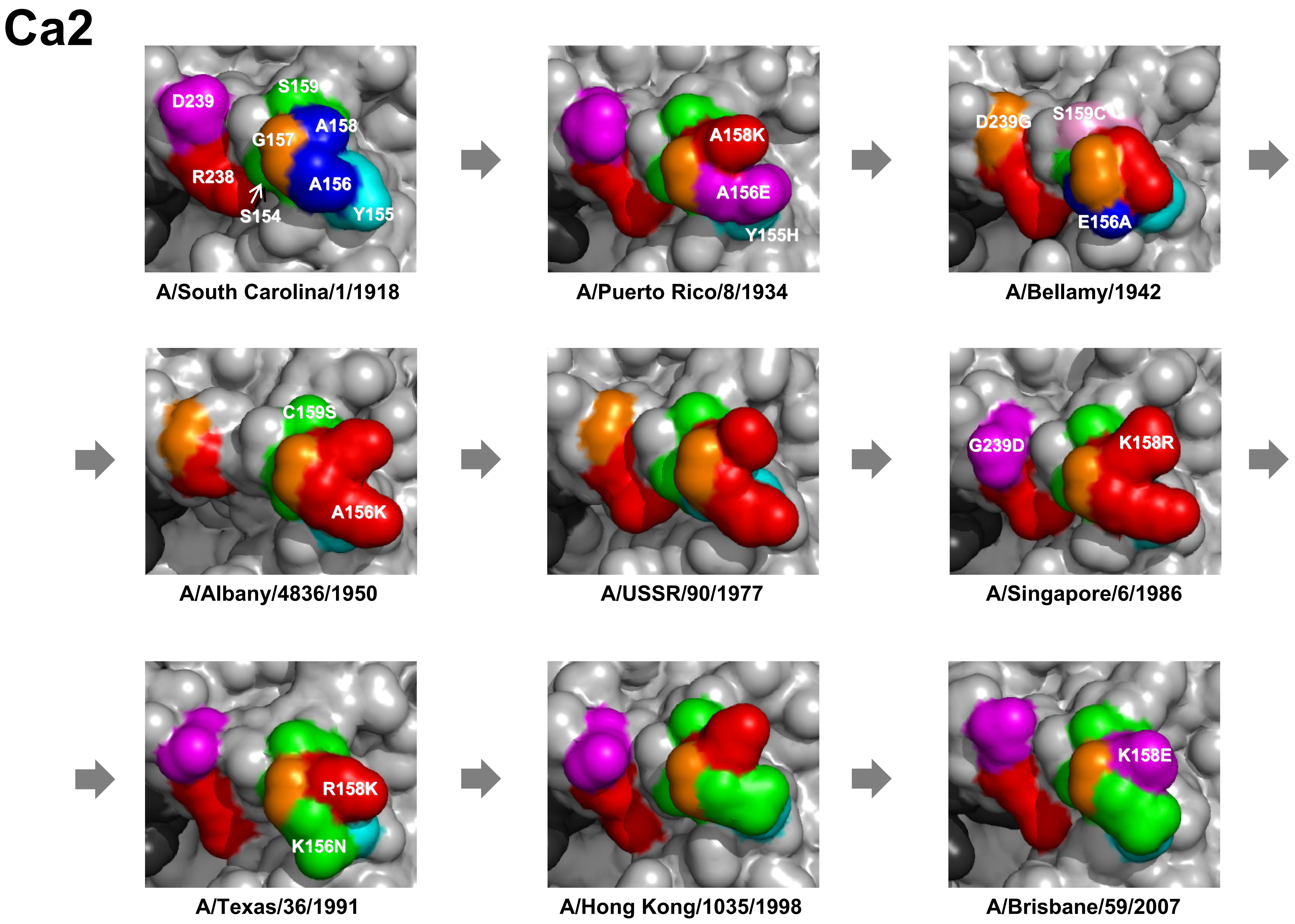
Ca2
D239
S159
A158
G157
R238
A156
S154
Y155
A/South Carolina/1/1918
A158K
A156E
Y155H
A/Puerto Rico/8/1934
D239G
S159C
E156A
A/Bellamy/1942
C159S
A156K
A/Albany/4836/1950
A/USSR/90/1977
G239D
K158R
A/Singapore/6/1986
R158K
K156N
A/Texas/36/1991
A/Hong Kong/1035/1998
K158E
A/Brisbane/59/2007

Cb

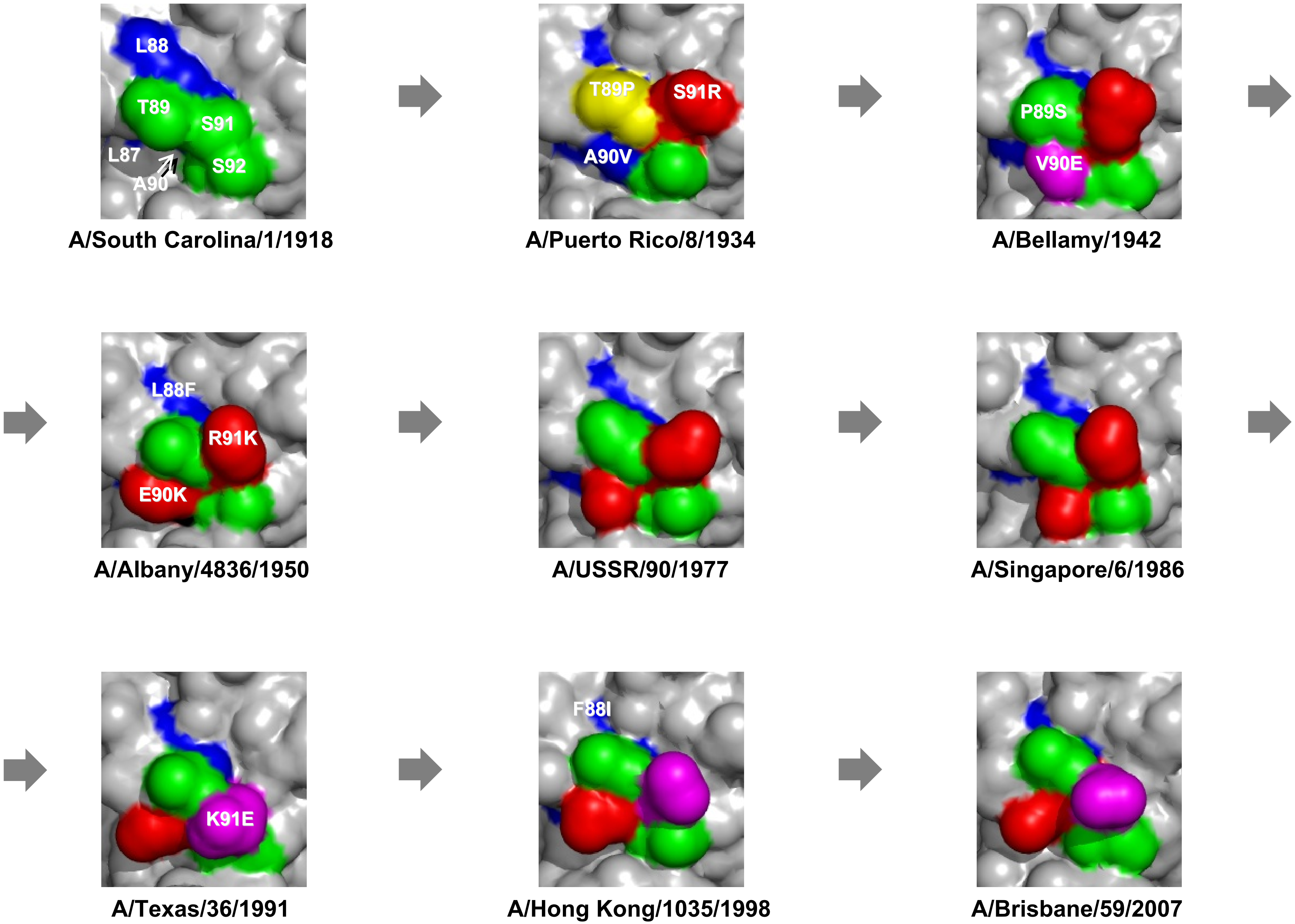
L88
T89
S91
L87
A90
S92
A/South Carolina/1/1918
T89P
S91R
A90V
A/Puerto Rico/8/1934
P89S
V90E
A/Bellamy/1942
L88F
R91K
E90K
A/Albany/4836/1950
A/USSR/90/1977
A/Singapore/6/1986
K91E
A/Texas/36/1991
F88I
A/Hong Kong/1035/1998
A/Brisbane/59/2007
